# Endovascular treatment in comatose patients with anterior circulation ischemic stroke

**DOI:** 10.3389/fneur.2025.1524262

**Published:** 2025-04-01

**Authors:** Wouter M. Sluis, Simone M. Uniken Venema, Anouk van der Hoorn, Joseph C. J. Bot, Wim H. van Zwam, Jeannette Hofmeijer, H. Bart van der Worp

**Affiliations:** ^1^Department of Neurology and Neurosurgery, UMC Utrecht Brain Center, University Medical Center Utrecht, Utrecht University, Utrecht, Netherlands; ^2^Department of Radiology, University Medical Center Utrecht, Utrecht University, Utrecht, Netherlands; ^3^Department of Radiology, Medical Imaging Center (MIC), University Medical Center Groningen, Groningen, Netherlands; ^4^Department of Radiology and Nuclear Medicine, Amsterdam University Medical Centers, Amsterdam, Netherlands; ^5^Department of Radiology and Nuclear Medicine, Maastricht University Medical Center, Maastricht, Netherlands; ^6^Department of Neurology, Rijnstate Hospital, Arnhem, Netherlands; ^7^Department of Clinical Neurophysiology, University of Twente, Enschede, Netherlands

**Keywords:** stroke, MR CLEAN, coma, endovascular treatment (EVT), ischemic

## Abstract

**Background:**

Coma in the first hours after anterior circulation ischemic stroke is rare. We aimed to assess the causes of coma and outcomes after endovascular thrombectomy (EVT) in this relatively unexplored subgroup of patients.

**Materials and methods:**

We used data from the MR CLEAN Registry, a prospective, multicenter, observational cohort study of patients treated with EVT in the Netherlands between March 2014, and December 2018. We included patients with anterior circulation ischemic stroke treated within 6.5 h of symptom onset and assessed frequency and causes of coma, defined as a score of 8 or lower on the Glasgow Coma Scale. Patients with a posterior circulation stroke were excluded. The primary outcome was the score on the modified Rankin Scale at 90 days. We compared outcomes of comatose and non-comatose patients with logistic regression.

**Results:**

Fifty-two (1%) of 4,869 patients were comatose. The main causes of coma were bilateral ischemia, a post-ictal state after an epileptic seizure, and respiratory insufficiency. Comatose patients were less likely to receive intravenous thrombolysis (54% vs. 73%; *p* = 0.004) and onset-to-groin times were longer (226 vs. 199 min; *p* = 0.012). Patients with coma had poorer functional outcomes (adjusted common odds ratio (OR), 2.73; 95%CI: 1.45–5.13) and more frequently died within 90 days (adjusted OR, 2.95; 95%CI: 1.47–5.90).

**Conclusion:**

Bilateral ischemia, a post-ictal state after an epileptic seizure and respiratory insufficiency are common causes of coma in patients with anterior circulation ischemic stroke treated with EVT. These patients have a high risk of death or dependency at 90 days.

## Introduction

About one fifth of patients with acute ischemic stroke caused by a proximal arterial occlusion in the anterior circulation has a reduced consciousness on admission in the first 6 h of stroke onset (1), but coma, defined as a score on the Glasgow Coma Scale (GCS) of 8 or lower (2), is rare (3). In the first hours of anterior circulation ischemic stroke, coma may be caused by simultaneous involvement of both hemispheres, or by new ischemia in the hemisphere contralateral to a large previous hemispheric lesion. In about one third of the patients no underlying cause is found (3, 4). Coma could also be caused by stroke-induced epileptic seizures or by other causes than the stroke itself, such as hypoglycaemia or severe arterial hypotension (5).

Coma is not a contraindication to endovascular thrombectomy (EVT) in patients with anterior circulation ischemic stroke due to a large vessel occlusion (LVO) (6, 7), but the frequency of this condition and the outcomes after EVT are unknown. We therefore aimed to assess the frequency of coma in patients with ischemic stroke caused by a proximal occlusion in the anterior circulation, the causes of such coma, and the outcomes after EVT.

## Materials and methods

### Study protocol and population

We used data from the Multicenter Randomized Controlled Trial of Endovascular Treatment for Acute Ischemic Stroke in the Netherlands (MR CLEAN) Registry - a prospective, multicenter, observational study of patients with acute ischemic stroke treated with EVT in routine clinical practice in the Netherlands in 17 centers (8). The current study is based on data from patients included in the Registry between March 16, 2014, and December 31, 2018. Inclusion criteria for the present analysis were: age ≥ 18 years; a clinical diagnosis of acute ischemic stroke with a proximal arterial occlusion in the anterior circulation (internal carotid artery, middle cerebral artery [M1 and M2], or anterior cerebral artery [A1 and A2]), demonstrated with computed tomography angiography, magnetic resonance angiography, or digital subtraction angiography; and EVT initiated within 6.5 h of symptom onset or last seen well. We adhered to the RECORD guidelines (9). The checklist is attached as a Supplementary Table S1. The study protocol of the MR CLEAN Registry was reviewed by the Erasmus University Medical Center Ethics Committee, which served as the central review board for all participating centers. The requirement for written informed consent was waived. Patients or their representatives were provided with information on the study orally and in writing and were given the opportunity to refuse participation.

### Data collection

Local investigators collected clinical and demographic data from patient records and assessed the score on the modified Rankin Scale (mRS) at 90 days (+/− 14 days).

Baseline imaging characteristics, including occlusion site, the Alberta Stroke Program Early CT Score (ASPECTS), and collateral status (10, 11), the extended treatment in cerebral infarction (eTICI) score after EVT (12), and follow-up imaging were assessed by the MR CLEAN Registry imaging core laboratory, blinded to clinical findings. Complications after treatment were assessed by a central serious adverse events adjudication committee, and included recurrent ischemic stroke, pneumonia, symptomatic intracranial hemorrhage (sICH, defined according to the Heidelberg Bleeding Classification) (13), and stroke progression (defined as neurological deterioration with an increase of ≥4 points on the NIHSS and follow-up imaging compatible with ischemia in the same territory without another underlying cause of deterioration). If no follow-up imaging was performed this was defined as neurological deterioration with an unknown cause.

As the GCS was not routinely collected in the Registry, written clinical information was evaluated for the GCS for all patients with a baseline score of 2 or higher on the level of consciousness question (item 1a) of the NIHSS. Patients were classified as comatose if the score on GCS derived from the written clinical information was 8 or lower. When patients were intubated and sedated for another reason (e.g., respiratory insufficiency due to a non-neurological cause), they were not counted as comatose if the GCS before intubation was known and above 8. If a pre-intubation GCS was not available, these patients were not considered comatose. The written clinical information of comatose patients was used to determine causes of coma.

### Outcomes

The primary outcome measure was the score on the mRS at 90 (± 14) days. Secondary outcome measures were mortality at 90 days and futile recanalization, defined as functional dependence or death (mRS score, ≥ 3) at 90 days despite successful recanalization (eTICI ≥2b) at the end of EVT.

### Statistical analysis

The frequency and causes of coma are described with descriptive statistics. Comparisons between baseline characteristics of patients who were comatose and those who were not were made with *χ*2, Student’s t or Mann–Whitney U test, where appropriate. We used multivariable (ordinal) logistic regression to calculate adjusted (common) odds ratios (a(c)OR) for all outcomes and adjusted for age, pre-stroke score on the mRS, NIHSS at baseline, onset-to-groin time, history of diabetes, history of atrial fibrillation, and treatment with intravenous thrombolysis. For the multivariable regression analyses, multiple imputation was performed for predictor values with missing data. A sensitivity analysis was performed without using multiple imputation for missing data. Statistical significance was defined as a *p* value <0.05. All statistical analyses were performed with R studio version 1.3.1056. (Rstudio PBC).

## Results

A total of 4,869 patients were included in this analysis (Supplementary Figure S1). Fifty-two (1%) patients were comatose before EVT. In 20 patients (39%) the cause of coma was a radiologically apparent bilateral ischemic lesion. Twelve patients had acute ischemic stroke in the presence of a previous cortical ischemic stroke in the contralateral hemisphere and eight had acute bilateral ischemic stroke, of whom six had a bilateral large vessel occlusion (LVO). Only one patient with a bilateral LVO was treated with EVT on both sides, which was only successful for one side. Of the remaining comatose patients, nine (17%) were respiratory insufficient, five (10%) were in a post-ictal state after a seizure, two (4%) were hypotensive due to cardiogenic shock, and the other patients were either hypothermic (1 patient, 2%), intoxicated with alcohol (1 patient, 2%), post-anoxic after cardiac arrest (1 patient, 2%) or in septic shock (1 patient, 2%). In twelve patients (23%) no clear cause of coma could be determined. Six of these patients eventually died of space-occupying edema, which was not visible on non-contrast CT before EVT. None of these patients was treated with decompressive surgery.

Patients who were comatose were comparable to non-comatose patients with respect to age, sex, medication, and comorbidities but were more frequently functionally dependent before the stroke (33% vs. 13%, *p* < 0.001), had a higher NIHSS (25 vs. 15, *p* < 0.001), more often a stroke of the left hemisphere (75% vs. 53%, *p* = 0.002), or a carotid-T occlusion [internal carotid artery (ICA) + middle cerebral artery (MCA), 39% vs. 19% (*p* = 0.001)]. Fewer comatose patients were given IVT (54% vs. 73%, *p* = 0.003) and they had worse ASPECTS and collateral scores (Table 1). Two comatose patients (3.8%) had a very low ASPECTS score (0 through 2), which was slightly higher than the group of patients without coma (*n* = 51, 1.1%).

**Table 1:** Baseline characteristics of patients with or without coma at hospital admission.

Characteristics*	Not comatose *n* = 4,817	Comatose *n* = 52	*p*-value
Clinical characteristics			
Age, years (median; IQR)	72.0 (62.0–81.0)	73.5 (61.5–82.0)	*p* = 0.742
Sex (female)	2,287 (47.5)	31 (59.6)	*p* = 0.109
Pre-stroke functional dependency †	600 (12.8)	16 (32.7)	***p* < 0.001**
NIHSS (median; IQR)	15.0 (11.0–19.0)	25.0 (20.8–30.0)	***p* < 0.001**
Left hemisphere	2,356 (52.5)	39 (75.0)	***p* = 0.002**
In-hospital stroke	501 (10.4)	10 (19.2)	*p* = 0.118
Treatment with IVT	3,514 (73.3)	28 (53.8)	***p* = 0.003**
Systolic BP (mean; SD)	150.1 (25.5)	145.9 (35.3)	*p* = 0.237
Laboratory characteristics			
INR (median: IQR)	1.0 (1.0–1.1)	1.1 (1.0–1.3)	***p* = 0.008**
CRP, mg/L (median; IQR)	4.9 (2.0–11.0)	4.0 (2.0–17.4)	*p* = 0.846
Glucose, mmol/L (median; IQR)	6.8 (5.9–8.1)	8.0 (6.6–10.3)	***p* = 0.001**
Imaging characteristics			
ICA-T occlusion	888 (19.2)	20 (39.2)	***p* = 0.001**
ASPECTS			***p* = 0.003**
0–4	193 (4.1)	7 (13.7)	
5–7	885 (19.0)	10 (19.6)	
8–10	3,590 (76.9)	34 (66.7)	
Collateral score^‡^			***p* = 0.001**
0	244 (5.4)	9 (18.0)	
1	1,653 (36.4)	18 (36.0)	
2	1756 (38.6)	18 (36.0)	
3	891 (19.6)	5 (10.0)	
Medical history			
Hypertension	2,513 (53.2)	27 (52.9)	*p* = 1.000
Atrial fibrillation	1,145 (24.1)	16 (30.8)	*p* = 0.339
Diabetes	809 (16.9)	10 (19.2)	*p* = 0.795
Hypercholesterolemia	1,448 (31.3)	14 (27.5)	*p* = 0.656
Ischemic stroke	850 (17.8)	16 (30.8)	***p* = 0.025**
Myocardial infarction	689 (14.6)	7 (13.5)	*p* = 0.980
Peripheral arterial disease	433 (9.2)	5 (9.6)	*p* = 1.000
Medication use			
Antiplatelet therapy	1,495 (31.4)	20 (40.0)	*p* = 0.252
DOAC	227 (4.7)	6 (12.0)	***p* = 0.040**
Coumarins	603 (12.6)	10 (19.6)	*p* = 0.201
Heparin	146 (3.1)	6 (12.0)	***p* = 0.001**
Statins	1706 (36.2)	20 (41.7)	*p* = 0.524
Anti-hypertensive agents	2,623 (55.5)	28 (56.0)	*p* = 1.000

*All numbers are *n* (%) unless stated otherwise, ^†^modified Rankin scale of 3 or higher, ^‡^0 = absent collaterals; 1 = less than 50% filling of occluded area with collaterals; 2 = more than 50 but less than 100% filling of occluded area; 3 = 100% filling of occluded area.

IQR = interquartile range, NIHSS = national institute of health stroke scale, BP = blood pressure, SD = standard deviation, IVT = intravenous thrombolysis, INR = international normalized ratio, CRP = c-reactive protein, ICA = internal carotid artery, ICA-T = occlusion of the top of the internal carotid artery and proximal middle cerebral artery, M1/M2 = first and second segment of the middle cerebral artery, ASPECTS = Alberta Stroke Program Early CT Score, DOAC = direct oral anti-coagulant.Bold values indicate statistical significance.

Onset-to-groin times were longer for comatose patients (225 vs. 199 min, *p* = 0.012) and treatment was more often performed under general anesthesia (61% vs. 23% *p* < 0.001). The NIHSS at 24 to 48 h was higher in the comatose group (21 vs. 9, *p* < 0.001) and comatose patients were more often diagnosed with stroke progression (19% vs. 9%, *p* = 0.015) (Tables 2, 3).

**Table 2:** Treatment characteristics and complications of comatose and non-comatose patients.

	Not comatose *n* = 4,817	Comatose *n* = 52	*p*-value
Treatment characteristics*			
Onset to groin (mean; SD)^†^	199.0 (74.4)	225.3 (78.0)	***p* = 0.012**
Duration of procedure (mean; SD)^†^	62.2 (34.4)	65.8 (40.1)	*p* = 0.487
Onset to end of procedure (mean; SD)^†^	253.2 (81.8)	282.0 (78.3)	***p* = 0.014**
General anesthesia	1,057 (23.3)	30 (61.2)	***p* < 0.001**
Successful recanalization (TICI 2b-3)	2,976 (64.4)	39 (78.0)	*p* = 0.06
NIHSS after treatment (median; IQR)^‡^	9.0 (3.0–16.0)	21.0 (15.0–25.0)	***p* < 0.001**

^*^All numbers are *n* (%) unless stated otherwise, ^†^in minutes, ^‡^NIHSS measured 24 h-48 h post EVT.

SD = standard deviation, TICI **=** thrombolysis in cerebral infarction scale, NIHSS = national institute of health stroke scale, mRS = modified Rankin scale. Bold values indicate statistical significance.

**Table 3:** Complications in comatose and non-comatose patients.

	Not comatose *n* = 4,817	Comatose *n* = 52	*p* value
Pneumonia*	491 (10.2)	6 (11.5)	*p* = 0.929
Intracranial hemorrhage	278 (5.8)	6 (11.5)	*p* = 0.142
Recurrent ischemic stroke	73 (1.5)	2 (3.8)	*p* = 0.429
Stroke progression	416 (8.6)	10 (19.2)	***p* = 0.015**

^*^All numbers are *n* (%) unless stated otherwise.Bold values indicate statistical significance.

**Figure 1:**
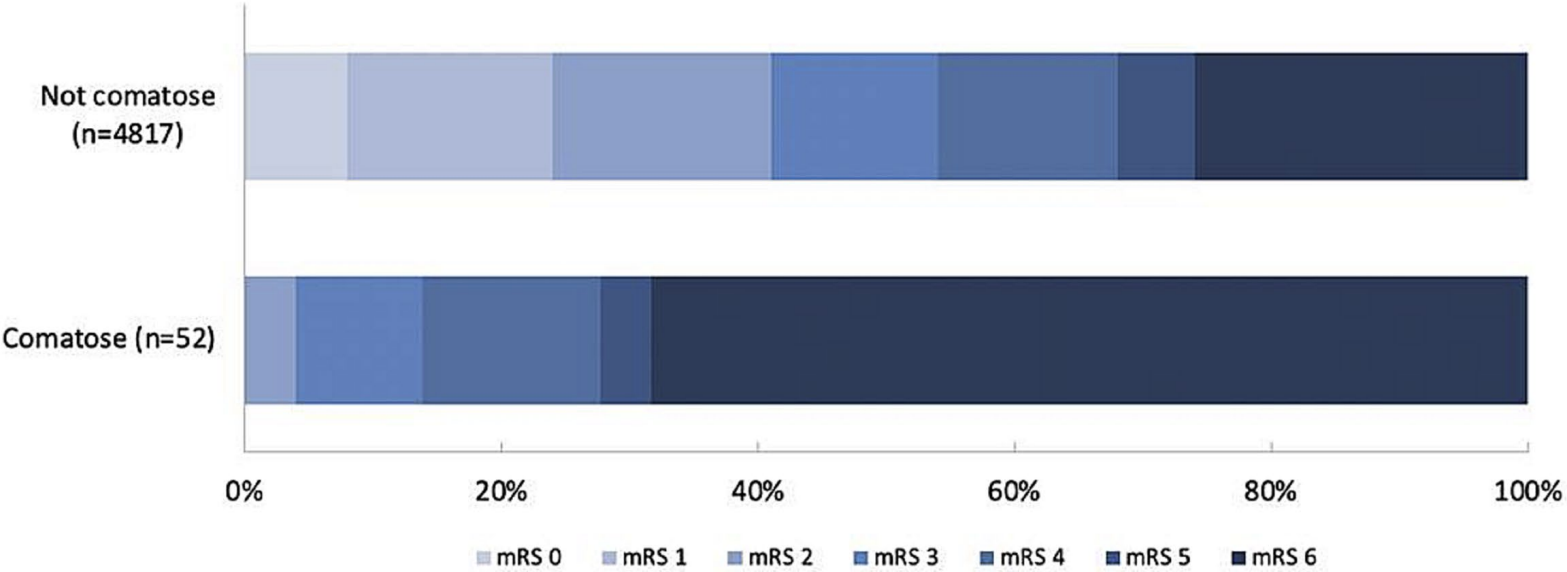
90-day mRS of patients with or without coma. mRS = modified Rankin Scale.

After adjustment for confounders, comatose patients died more often within 90 days (69% vs. 26%; aOR 2.95; 95%CI:1.47–5.90; Tables 1, 4; Figure 1), with a median time to death of 3 days (IQR 2–7).

**Table 4:** Outcome measures in comatose and non-comatose patients.

	Not comatose *n* = 4,817	Comatose *n* = 52	aOR (95%CI)
mRS at 90 days (median; IQR)	3.0 (2.0–6.0)	6.0 (4.0–6.0)	6.11 (3.42–10.90)^††^ 2.73 (1.45–5.13)^††,^*
90-day mortality	1,230 (26.4)	35 (68.6)	5.77 (3.22–10.34) 2.95 (1.47–5.90)*
Futile recanalization^†^	1,473 (32.6)	36 (73.5)	1.24 (0.79–1.95) 0.82 (0.51–1.31)*

*Adjusted for age, pre-stroke mRS, NIHSS at baseline, duration of onset to groin time, history of diabetes, history of atrial fibrillation and the use of IV thrombolytics. ^†^Unfavorable outcome (mRS ≥ 3) despite successful recanalization, ^††^adjusted Common Odds Ratio (aCOR).

aOR = adjusted odds ratio, mRS = modified Rankin Scale, IQR = interquartile range.

Mortality was highest in comatose patients with a bilateral ischemic lesion (70%) or a non-neurological cause of coma (71%) and lowest in the post-ictal patients (40%). Comatose patients had a worse functional outcome at 90 days (acOR for a shift on the mRS towards a worse functional outcome: 2.73; 95%CI: 1.45–5.13; Table 4; Figure 1) and just two patients (4%) were functionally independent at 90 days: one patient in a post-ictal state after a seizure and one with an unknown cause of coma. Recanalization was futile in 74% of comatose patients. The sensitivity analysis without imputed data yielded similar results (Supplementary Table S2).

## Discussion

In this study, patients with ischemic stroke caused by a proximal occlusion in the anterior circulation who were comatose within 6.5 h of stroke onset had a very high risk of death or dependency at 90 days after EVT, even with angiographic recanalization. The most frequent cause of coma was an ischemic lesion in the hemisphere contralateral to the occlusion.

Acute bilateral large vessel occlusion occurs in less than 1% of patients with ischemic stroke. The prognosis is generally poor, as was the case in our six patients (4). For unknown reasons, only one of them was treated on both sides and recanalization only occurred on one side. We therefore do not know whether outcomes would have been better if patients with bilateral occlusions had bilateral recanalization. Contralateral recurrent stroke occurred in 7% of patients in The Copenhagen Stroke Study and was also associated with a worse functional outcome compared to ipsilateral recurrence (14).

Most patients with a systemic non-neurological cause of coma had signs of severe metabolic, respiratory or hemodynamic dysregulation. Hypotension, hypoxia, and infections after stroke have been associated with a worse prognosis (15–17). In about one quarter of comatose patients, no cause of reduced consciousness was identified, which is comparable to a previous study in which no cause of coma was found (3). Remarkably, in our study more than half of the patients with coma of unidentified cause died of space-occupying edema in the next days, without signs of mass effect on non-contrast CT before EVT, none of the patients in our study were treated with decompressive surgery, as opposed to around 40% in a previous MR CLEAN study (18). With the exception of one patient, all these patients primarily presented to the intervention center, so most imaging was directly prior to EVT. Space-occupying edema is a well-known cause of coma in patients with unilateral anterior circulation stroke, but clear clinical signs of mass effect generally occur after the first few hours, and only in one third of the cases within 24 h (19, 20). In a study of patients with middle cerebral artery infarction caused by an M1 occlusion, 15 of the 24 patients (63%) who eventually developed ‘malignant’ space-occupying edema had a reduced level of consciousness in the first 6 hours of stroke onset, but the proportion of patients with coma in this study is not known (21).

The finding that comatose patients were less frequently treated with IVT and had longer door-to-groin times could suggest that treating physicians are hesitant to treat comatose patients, possibly due to a lack of evidence on the efficacy of reperfusion therapy in this subgroup of patients. Another explanation could be a greater diagnostic delay, and therefore missing the time window for treatment with IVT, or suspected comorbidity with a suspected increased risk hemorrhage. In a study on IVT in patients with ischemic stroke and a decreased consciousness, IVT seemed beneficial, but this study was just as the present study not randomized and retrospective in design, possibly causing selection bias (22).

The current study is the first to report causes of coma and outcomes of EVT in patients with anterior circulation ischemic stroke, but there are limitations to consider. First, this is a retrospective analysis in a prospective registry of patients who were treated with EVT. We therefore cannot provide information on comatose patients with anterior circulation ischemic stroke who were not treated with EVT, for example because they were considered by the clinician as too severe for curative treatment. Second, causes of coma were determined retrospectively based on written clinical information, which may be insufficient to accurately reflect the actual causes of coma. EEG was not routinely performed and the presence or absence of a fetal posterior communicating artery was not assessed. Third, the group of patients with coma was very small compared to the non-comatose group and therefore the uncertainty of our estimates is considerable. Fourth, patients included in the MR CLEAN Registry were treated early in the era of EVT. Since then, devices and skills have improved which could positively influence outcomes of comatose patients. Nevertheless, our data strongly suggest that the risk of a poor outcome after EVT is high in patients who are comatose on admission is, especially in patients with bilateral ischemic stroke. Further research is needed to assess the benefit of EVT in patients without a known cause of coma.

## Conclusion

Patients with anterior circulation ischemic stroke who are comatose before endovascular thrombectomy in the first 6.5 h often have ischemic lesions in both hemispheres or an underlying metabolic or hemodynamic dysregulation as the cause of their decreased consciousness. These patients have a very poor prognosis despite successful EVT.

## Data Availability

The data analyzed in this study is subject to the following licenses/restrictions: the dataset can be obtained upon reasonable request via the MR CLEAN writing committee. Requests to access these datasets should be directed to https://www.mrclean-trial.org/.

## MR CLEAN Registry Investigators

A list of all MR CLEAN Registry Investigators is provided in the Supplementary material.
