# Supplementary material for: Endovascular treatment in comatose patients with anterior circulation ischemic stroke
Source: Front Neurol. 2025 Apr 1;16:1524262. doi: 10.3389/fneur.2025.1524262 (PMC11997383; doi:10.3389/fneur.2025.1524262)
Supplement: Supplementary file 1 [file Table_1.docx]

**SUPPLEMENT**

**Endovascular treatment in comatose patients with anterior circulation ischemic stroke**

Wouter M. Sluis, Simone M. Uniken Venema, Anouk van der Hoorn, Joseph C.J. Bot, Wim H. van Zwam, Jeannette Hofmeijer, H. Bart van der Worp

|  | **Title** | **Page** |
| --- | --- | --- |
| **Table S1** | RECORD checklist | 2 |
| **Figure S1** | Flowchart of patient inclusion | 12 |
| **Table S2** | Sensitivity analysis without imputed data | 13 |
| **Table S3** | Predictor and outcome values with a missing percentage of 1% or higher | 16 |
| **Table S4** | MR CLEAN Registry Investigators | 17 |

**Table S1.** The RECORD statement – checklist of items, extended from the STROBE statement, that should be reported in observational studies using routinely collected health data**.**

|  | **Item No.** | **STROBE items** | **Location in manuscript where items are reported** | **RECORD items** | **Location in manuscript where items are reported** |
| --- | --- | --- | --- | --- | --- |
| **Title and abstract** | | |  |  |  |
|  | 1 | (a) Indicate the study’s design with a commonly used term in the title or the abstract (b) Provide in the abstract an informative and balanced summary of what was done and what was found | Page 3 | RECORD 1.1: The type of data used should be specified in the title or abstract. When possible, the name of the databases used should be included.    RECORD 1.2: If applicable, the geographic region and timeframe within which the study took place should be reported in the title or abstract.    RECORD 1.3: If linkage between databases was conducted for the study, this should be clearly stated in the title or abstract. | Page 3  Page 3  n.a. |
| **Introduction** | | |  |  |  |
| Background rationale | 2 | Explain the scientific  background and rationale for the investigation being reported | Page 5 |  |  |
| Objectives | 3 | State specific objectives, including any prespecified hypotheses | Page 5 |  |  |
| **Methods** | | |  |  |  |
| Study Design | 4 | Present key elements of study design early in the paper | Page 6 |  |  |
| Setting | 5 | Describe the setting, locations, and relevant dates, including periods of recruitment, exposure, follow-up, and data collection | Page 6 |  |  |

| Participants | 6 | 1. *Cohort study* - Give the eligibility criteria, and the sources and methods of selection of participants. Describe methods of follow-up   *Case-control study* - Give the eligibility criteria, and the sources and methods of case ascertainment and control selection. Give the rationale for the choice of cases and controls *Cross-sectional study* - Give the eligibility criteria, and the sources and methods of selection of participants     1. *Cohort study* - For matched studies, give matching criteria and number of exposed and unexposed   *Case-control study* - For matched studies, give matching criteria and the number of controls per case | Page 6  n.a.  n.a.  n.a. | RECORD 6.1: The methods of study population selection (such as codes or algorithms used to identify subjects) should be listed in detail. If this is not possible, an explanation should be provided.    RECORD 6.2: Any validation studies of the codes or algorithms used to select the population should be referenced. If validation was conducted for this study and not published elsewhere, detailed methods and results should be provided.    RECORD 6.3: If the study involved linkage of databases, consider use of a flow diagram or other graphical display to demonstrate the data linkage process, including the number of individuals with linked data at each stage. | Page 6  n.a.  n.a. |
| --- | --- | --- | --- | --- | --- |
| Variables | 7 | Clearly define all outcomes, exposures, predictors, potential confounders, and effect modifiers. Give diagnostic criteria, if applicable. | Page 7 | RECORD 7.1: A complete list of codes and algorithms used to classify exposures, outcomes, confounders, and effect modifiers should be provided. If these cannot be reported, an explanation should be provided. | n.a. |
| Data sources/ measurement | 8 | For each variable of interest, give sources of data and details of methods of assessment (measurement).  Describe comparability of assessment methods if there is  more than one group | Page 6+7 |  |  |

| Bias | 9 | Describe any efforts to address potential sources of bias | n.a. |  |  |
| --- | --- | --- | --- | --- | --- |
| Study size | 10 | Explain how the study size was arrived at | n.a. |  |  |
| Quantitative variables | 11 | Explain how quantitative variables were handled in the analyses. If applicable, describe which groupings were chosen,  and why | Page 7+8 |  |  |
| Statistical methods | 12 | (a) Describe all statistical methods, including those used to control for confounding (b) Describe any methods used to examine subgroups and interactions   1. Explain how missing data were addressed 2. *Cohort study* - If applicable, explain how loss to follow-up was addressed   *Case-control study* - If applicable, explain how matching of cases and controls was addressed  *Cross-sectional study* - If applicable, describe analytical methods taking account of sampling strategy   1. Describe any sensitivity analyses | Page 7+8  Page 8  n.a.  n.a. |  |  |
| Data access and cleaning methods |  | .. |  | RECORD 12.1: Authors should describe the extent to which the investigators had access to the database population used to create the study population. | Page 8 |

|  |  |  |  | RECORD 12.2: Authors should provide information on the data cleaning methods used in the study. | n.a. |
| --- | --- | --- | --- | --- | --- |
| Linkage |  | .. |  | RECORD 12.3: State whether the study included person-level,  institutional-level, or other data linkage across two or more databases. The methods of linkage and methods of linkage quality evaluation should be provided. | Page 8 |
| **Results** | | | | | |
| Participants | 13 | 1. Report the numbers of individuals at each stage of the study (*e.g.*, numbers potentially eligible, examined for eligibility, confirmed eligible, included in the study, completing follow-up, and analysed) 2. Give reasons for nonparticipation at each stage. (c) Consider use of a flow diagram | Page 9  Page 9 and flow-diagram  Supplemental figure S1 | RECORD 13.1: Describe in detail the selection of the persons included in the study (*i.e.,* study population selection) including filtering based on data quality, data availability and linkage. The selection of included persons can be described in the text and/or by means of the study flow diagram. | Supplemental figure S1 |
| Descriptive data | 14 | 1. Give characteristics of study participants (*e.g.*, demographic, clinical, social) and information on exposures and potential   confounders   1. Indicate the number of participants with missing data for each variable of interest (c) *Cohort study* - summarise follow-up time (*e.g.*, average and total amount) | Page 9  Supplement table S4 |  |  |
| Outcome data | 15 | *Cohort study* - Report numbers of outcome events or summary measures over time  *Case-control study* - Report numbers in each exposure | Page 9 + 10  n.a. |  |  |

|  |  | category, or summary measures of exposure  *Cross-sectional study* - Report numbers of outcome events or summary measures | n.a. |  |  |
| --- | --- | --- | --- | --- | --- |
| Main results | 16 | (a) Give unadjusted estimates and, if applicable, confounderadjusted estimates and their precision (e.g., 95% confidence interval). Make clear which confounders were adjusted for and why they were included (b) Report category boundaries when continuous variables were categorized  (c) If relevant, consider translating estimates of relative risk into absolute risk for a meaningful time period | Page 10 |  |  |
| Other analyses | 17 | Report other analyses done—  e.g., analyses of subgroups and interactions, and sensitivity analyses | Page 10 |  |  |
| **Discussion** | | | | | |
| Key results | 18 | Summarise key results with reference to study objectives | Page 11 |  |  |
| Limitations | 19 | Discuss limitations of the study, taking into account sources of potential bias or imprecision. Discuss both direction and magnitude of any potential bias | Page 12 | RECORD 19.1: Discuss the  implications of using data that were not created or collected to answer the specific research question(s). Include discussion of misclassification bias, unmeasured confounding, missing data, and changing eligibility over time, as they pertain to the study being reported. | Page 12 |
| Interpretation | 20 | Give a cautious overall interpretation of results considering objectives, | Page 13 |  |  |
|  |  | limitations, multiplicity of analyses, results from similar studies, and other relevant evidence |  |  |  |
| Generalisability | 21 | Discuss the generalisability (external validity) of the study results | Page 13 |  |  |
| **Other Information** | | | | | |
| Funding | 22 | Give the source of funding and the role of the funders for the present study and, if applicable, for the original study on which the present article is based | Page 15 |  |  |
| Accessibility of protocol, raw data, and programming code |  | .. |  | RECORD 22.1: Authors should provide information on how to access any supplemental information such as the study protocol, raw data, or programming code. | Page 8 |

*Reference: Benchimol EI, Smeeth L, Guttmann A, Harron K, Moher D, Petersen I, Sørensen HT, von Elm E, Langan SM, the RECORD Working Committee. The REporting of studies Conducted using Observational Routinely-collected health Data (RECORD) Statement. *PLoS Medicine* 2015; in press.

*Checklist is protected under Creative Commons Attribution ([CC BY)](http://creativecommons.org/licenses/by/4.0/) license.

**Figure S1.** Flowchart of patient inclusion

**
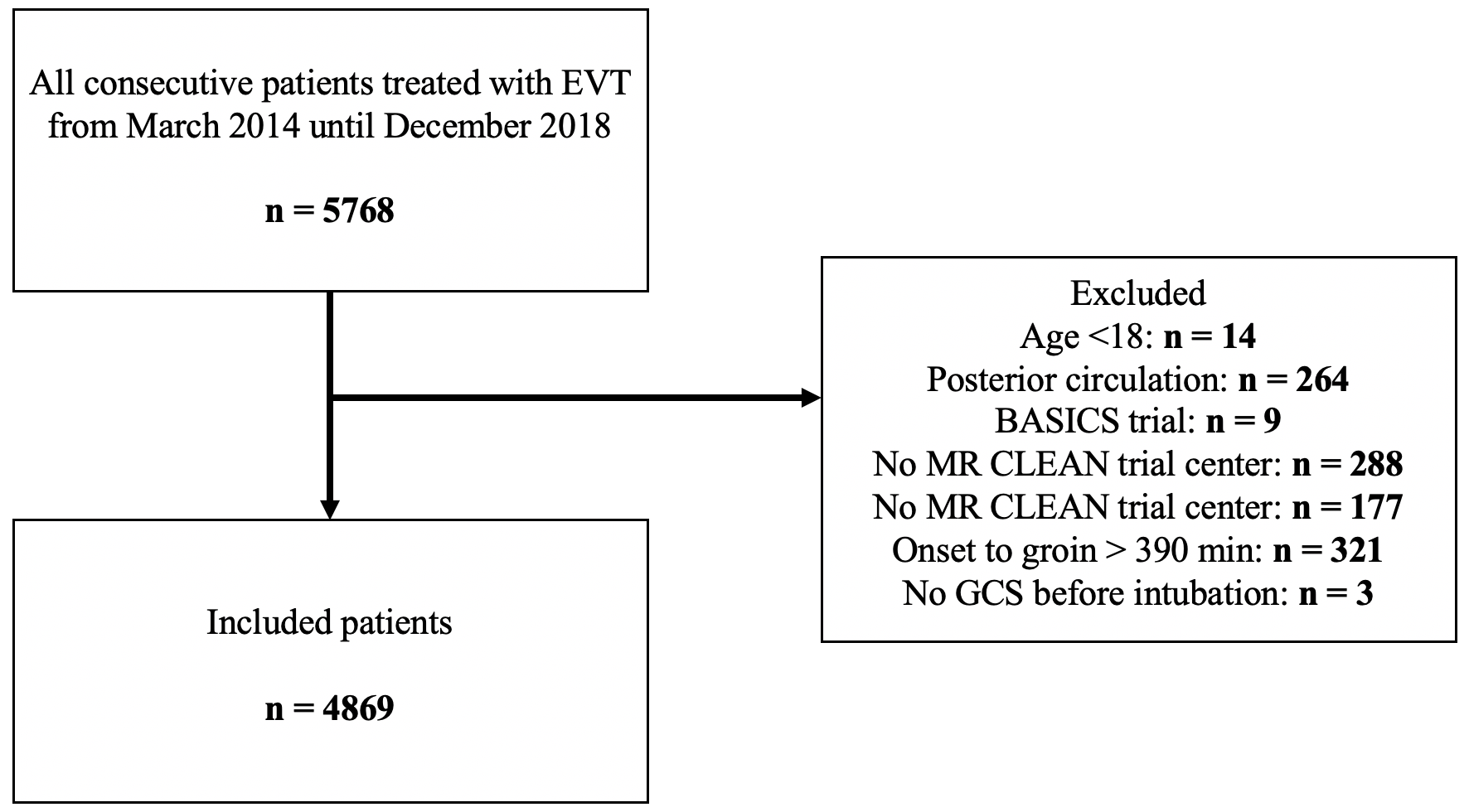
**

**Table S2.** Outcome measures in comatose and non-comatose patients, sensitivity analysis without imputed data

|  | **Not comatose**  **n=** 4817 | **Comatose**  **n=** 52 | **OR** (95%CI) |
| --- | --- | --- | --- |
| mRS at 90 days (median; IQR)  90-day mortality  Futile recanalization ^†^ | 3.0 (2.0-6.0)  1230 (26.4)  1473 (32.6) | 6.0 (4.0-6.0)  35 (68.6)  36 (73.5) | 6.46 (3.63-11.48**)**  2.51 (1.31-4.80)*  6.11 (3.37-11.08)  2.37 (1.12-5.01)*  1.21 (0.77-1.91)  0.75 (0.46-1.24)* |

* adjusted for age, pre-stroke mRS, NIHSS at baseline, duration of onset to groin time, history of diabetes, history of atrial fibrillation and the use of IV-thrombolytics. ^†^ Unfavorable outcome (mRS > 3) despite complete recanalization

**Abbreviations:** OR = odds ratio, mRS = modified Rankin Scale, IQR = interquartile range

**Table S3**. Predictor and outcome values with a missing percentage of 1% or higher

| **Predictors** | **Missing values (n%)** |  |
| --- | --- | --- |
| Death at 90 days  mRS at 90 days  Pre-stroke mRS  Onset to groin time  NIHSS at baseline  Medical history of atrial fibrillation | 151 (3.1)  151 (3.1)  131 (2.7)  90 (1.8)  69 (1.4)  65 (1.3) | |

**Abbreviations:** mRS = modified Rankin Scale, NIHSS = national institute of health stroke scale

**Table S4.** MR CLEAN Registry Investigators

Executive committee

Diederik W.J. Dippel^1^;Aad van der Lugt^2^;Charles B.L.M. Majoie^3^;Yvo B.W.E.M. Roos^4^;Robert J. van Oostenbrugge^5,41^;Wim H. van Zwam^6,41^;Jelis Boiten^14^;Jan Albert Vos^8^

Study coordinators

Ivo G.H. Jansen^3^;Maxim J.H.L. Mulder^1,2^;Robert- Jan B. Goldhoorn^5,6,41^;Kars C.J. Compagne^2^;Manon Kappelhof^3^;Josje Brouwer^4^;Sanne J. den Hartog^1,2,40^;Wouter H. Hinsenveld ^5,6^;

Local principal investigators

Diederik W.J. Dippel^1^;Bob Roozenbeek^1^;Aad van der Lugt^2^;Adriaan C.G.M. van Es^2^;Charles B.L.M. Majoie^3^;Yvo B.W.E.M. Roos^4^;Bart J. Emmer^3^;Jonathan M. Coutinho^4^;Wouter J. Schonewille^7^;Jan Albert Vos^8^; Marieke J.H. Wermer^9^;Marianne A.A. van Walderveen^10^;Julie Staals^5,41^;Robert J. van Oostenbrugge^5,41^,Wim H. van Zwam^6,41^;Jeannette Hofmeijer^11^;Jasper M. Martens^12^;Geert J. Lycklama à Nijeholt^13^;Jelis Boiten^14^;Sebastiaan F. de Bruijn^15^;Lukas C. van Dijk^16^;H. Bart van der Worp^17^;Rob H. Lo^18^;Ewoud J. van Dijk^19^;Hieronymus D. Boogaarts^20^;J. de Vries^22^;Paul L.M. de Kort^21^; Julia van Tuijl^21^ ; Jo P. Peluso^26^;Puck Fransen^22^;Jan S.P. van den Berg^22^;Boudewijn A.A.M. van Hasselt^23^;Leo A.M. Aerden^24^;René J. Dallinga^25^;Maarten Uyttenboogaart^28^;Omid Eschgi^29^;Reinoud P.H. Bokkers^29^;Tobien H.C.M.L. Schreuder^30^;Roel J.J. Heijboer^31^;Koos Keizer^32^;Lonneke S.F. Yo^33^;Heleen M. den Hertog^22^;Tomas Bulut^35^; Paul J.A.M. Brouwers^34^.

Imaging assessment committee

Charles B.L.M. Majoie^3^(chair);Wim H. van Zwam^6,41^;Aad van der Lugt^2^;Geert J. Lycklama à Nijeholt^13^;Marianne A.A. van Walderveen^10^;Marieke E.S. Sprengers^3^;Sjoerd F.M. Jenniskens^27^;René van den Berg^3^;Albert J. Yoo^38^;Ludo F.M. Beenen^3^;Alida A. Postma^6,42^, Stefan D. Roosendaal^3^;Bas F.W. van der Kallen^13^;Ido R. van den Wijngaard^13^;Adriaan C.G.M. van Es^2^;Bart J. Emmer^,3^;Jasper M. Martens^12^; Lonneke S.F. Yo^33^;Jan Albert Vos^8^; Joost Bot^36^, Pieter-Jan van Doormaal^2^; Anton Meijer^27^;Elyas Ghariq^13^; Reinoud P.H. Bokkers^29^;Marc P. van Proosdij^37^;G. Menno Krietemeijer^33^;Jo P. Peluso^26^;Hieronymus D. Boogaarts^20^;Rob Lo^18^;Wouter Dinkelaar^2^Auke P.A. Appelman^29^;Bas Hammer^16^;Sjoert Pegge^27^;Anouk van der Hoorn^29^;Saman Vinke^20^.

Writing committee

Diederik W.J. Dippel^1^(chair);Aad van der Lugt^2^;Charles B.L.M. Majoie^3^;Yvo B.W.E.M. Roos^4^;Robert J. van Oostenbrugge^5,41^;Wim H. van Zwam^6,41^;Geert J. Lycklama à Nijeholt^13^;Jelis Boiten^14^;Jan Albert Vos^8^;Wouter J. Schonewille^7^;Jeannette Hofmeijer^11^;Jasper M. Martens^12^;H. Bart van der Worp^17^;Rob H. Lo^18^

Adverse event committee

Robert J. van Oostenbrugge^5,41^(chair);Jeannette Hofmeijer^11^;H. Zwenneke Flach^23^

Trial methodologist

Hester F. Lingsma^40^

Research nurses / local trial coordinators

Naziha el Ghannouti^1^;Martin Sterrenberg^1^;Wilma Pellikaan^7^;Rita Sprengers^4^;Marjan Elfrink^11^;Michelle Simons^11^;Marjolein Vossers^12^;Joke de Meris^14^;Tamara Vermeulen^14^;Annet Geerlings^19^;Gina van Vemde^22^;Tiny Simons^30^;Gert Messchendorp^28^;Nynke Nicolaij^28^;Hester Bongenaar^32^;Karin Bodde^24^;Sandra Kleijn^34^;Jasmijn Lodico^34^; Hanneke Droste^34^;Maureen Wollaert^5^;Sabrina Verheesen^5^;D. Jeurrissen^5^;Erna Bos^9^;Yvonne Drabbe^15^;Michelle Sandiman^15^;Nicoline Aaldering^11^;Berber Zweedijk^17^;Jocova Vervoort^21^;Eva Ponjee^22^;Sharon Romviel^19^;Karin Kanselaar^19^;Denn Barning^10^.

PhD / Medical students:

Esmee Venema^40^; Vicky Chalos^1,40^; Ralph R. Geuskens^3^; Tim van Straaten^19^;Saliha Ergezen^1^; Roger R.M. Harmsma^1^; Daan Muijres^1^; Anouk de Jong^1^;Olvert A. Berkhemer^1,3,6^;Anna M.M. Boers^3,39^; J. Huguet^3^;P.F.C. Groot^3^;Marieke A. Mens^3^;Katinka R. van Kranendonk^3^;Kilian M. Treurniet^3^;Manon L. Tolhuisen^3,39^;Heitor Alves^3^;Annick J. Weterings^3^,Eleonora L.F. Kirkels^3^,Eva J.H.F. Voogd^11^;Lieve M. Schupp^3^;Sabine L. Collette^28,29^;Adrien E.D. Groot^4^;Natalie E. LeCouffe^4^;Praneeta R. Konduri^39^;Haryadi Prasetya^39^;Nerea Arrarte-Terreros^39^;Lucas A. Ramos^39^.

List of affiliations

Department of Neurology^1^, Radiology^2^, Public Health^40^, Erasmus MC University Medical Center;

Department of Radiology and Nuclear Medicine^3^, Neurology^4^, Biomedical Engineering & Physics^39^,

Amsterdam UMC, location University of Amsterdam;

Department of Neurology^5^, Radiology & Nuclear Medicine^6^, Maastricht University Medical Center+; School for Cardiovascular Diseases Maastricht (CARIM)^41^; and MHeNs School for Mental Health and Neuroscience, Maastricht, the Netherlands^42^;

Department of Neurology^7^, Radiology^8^, Sint Antonius Hospital, Nieuwegein;

Department of Neurology^9^, Radiology^10^, Leiden University Medical Center;

Department of Neurology^11^, Radiology^12^, Rijnstate Hospital, Arnhem;

Department of Radiology^13^, Neurology^14^, Haaglanden MC, the Hague;

Department of Neurology^15^, Radiology^16^, HAGA Hospital, the Hague;

Department of Neurology^17^, Radiology^18^, University Medical Center Utrecht;

Department of Neurology^19^, Neurosurgery^20^, Radiology^27^, Radboud University Medical Center, Nijmegen;

Department of Neurology^21^, Radiology^26^, Elisabeth-TweeSteden ziekenhuis, Tilburg;

Department of Neurology^22^, Radiology^23^, Isala Klinieken, Zwolle;

Department of Neurology^24^, Radiology^25^, Reinier de Graaf Gasthuis, Delft;

Department of Neurology^28^, Radiology^29^, University Medical Center Groningen;

Department of Neurology^30^, Radiology^31^, Atrium Medical Center, Heerlen;

Department of Neurology^32^, Radiology^33^, Catharina Hospital, Eindhoven;

Department of Neurology^34^, Radiology^35^, Medisch Spectrum Twente, Enschede;

Department of Radiology^36^, Amsterdam UMC, Vrije Universiteit van Amsterdam, Amsterdam;
Department of Radiology^37^, Noordwest Ziekenhuisgroep, Alkmaar;

Department of Radiology^38^, Texas Stroke Institute, Texas, United States of America.
